# A Rare Pathological Couplet of Colocolic Intussusception Plus Cecal Bascule in a Young Adult: A Case Report

**DOI:** 10.7759/cureus.3430

**Published:** 2018-10-09

**Authors:** Auerilius E Hamilton, Angad Singh, Kirk K Austin, JooShik Shin, Jonathan S Hong

**Affiliations:** 1 Colorectal Surgery, Royal Prince Alfred Hospital, Sydney, AUS; 2 Pathology, Royal Prince Alfred Hospital, Sydney, AUS

**Keywords:** colorectal surgery, radiology, intussusception, caecal bascule

## Abstract

This report is of a rare case involving a 27-year-old female who presented to the hospital with the pathological couplet of colocolic intussusception and cecal bascule causing bowel obstruction. Up to the time of presentation to the hospital, this patient had not undergone a full investigation for a known iron deficiency, anemia. Subsequently, during the emergency admission and after having an operative surgical procedure, the patient was found to have both a congenitally malpositioned cecum and a benign colonic polyp-forming condition. The pertinent issues about this unusual case to be highlighted are its ambiguous clinical presentation; uncommon gender and age group for either condition; the simultaneous occurrence of dual anatomical anomalies; and the uncommon benign etiology of causes of bowel obstruction in adults.

## Introduction

Intussusception, a common pediatric abdominal emergency commonly seen in patients ≤ two years of age, occurs when a loop of bowel (the “intussusceptum”) telescopes into an adjoining segment (the “intussuscipiens”). An intussusception in an adult is rare, accounting for 1%-5% of all adult bowel obstructions, and colocolic intussusceptions are similarly rare at 5% of all cases of intussusceptions in all age groups [1]. Adults with this condition often have obstructive symptoms but may present with atypical abdominal pains, constipation, no hematochezia, or no vomiting. About 40% of cases of adult colocolic intussusceptions occur in the absence of a malignant lead-point [2]. A 10-year review by Barussaud et al. found that only 67% of intussusceptions involved a lead point at all and that ultimately surgical intervention is needed in most (72%) cases. Computed tomography (CT) was the preferred diagnostic imaging modality in most cases (81%) over colonoscopy, ultrasonography, and small bowel enteroclysis with a characteristic CT feature being the presence of a target sign or a sausage-shaped lesion [2-3].

Colonic volvulus is caused by the en masse torsion of a segment of the colon and its mesentery with the supplying vessels, leading to intestinal obstruction. Cecal volvulus is uncommon, accounting for about 15% of all cases of colonic volvulus. These can be classified into three types: the axial-torsion type where cecal rotation is in the axial plane; the loop-type, where there are both cecal rotation and inversion; or the bascule-type [4-5]. With a peak incidence of 55 years, cecal bascule is a rare occurrence with a slightly higher male predominance [5]. A cecal bascule, which represents about 10% of cecal volvuli, is characterized by a mispositioned cecum being folded in a cephalad (most common) or caudad direction with no torsion of its mesentery [4]. Its incidence is 2.8-7.1 per million people per year and several etiologies have been suggested, including congenital adhesions or distal colonic obstruction [5-7]. A cecal bascule can clinically present with such signs as cecal displacement to the upper or central abdomen, ileocecal valve displacement to the right upper quadrant, a transition zone between the cecum and ascending colon, and as reactive perihepatic free fluid [7-8]. There is no reported case of a combination of colocolic intussusception and cecal bascule simultaneously. This is an unusual pathological couplet of colonic pathologies with concomitant contralateral colonic ischemia that added more complexity to this rare clinical triad.

## Case presentation

**Figure 1:**
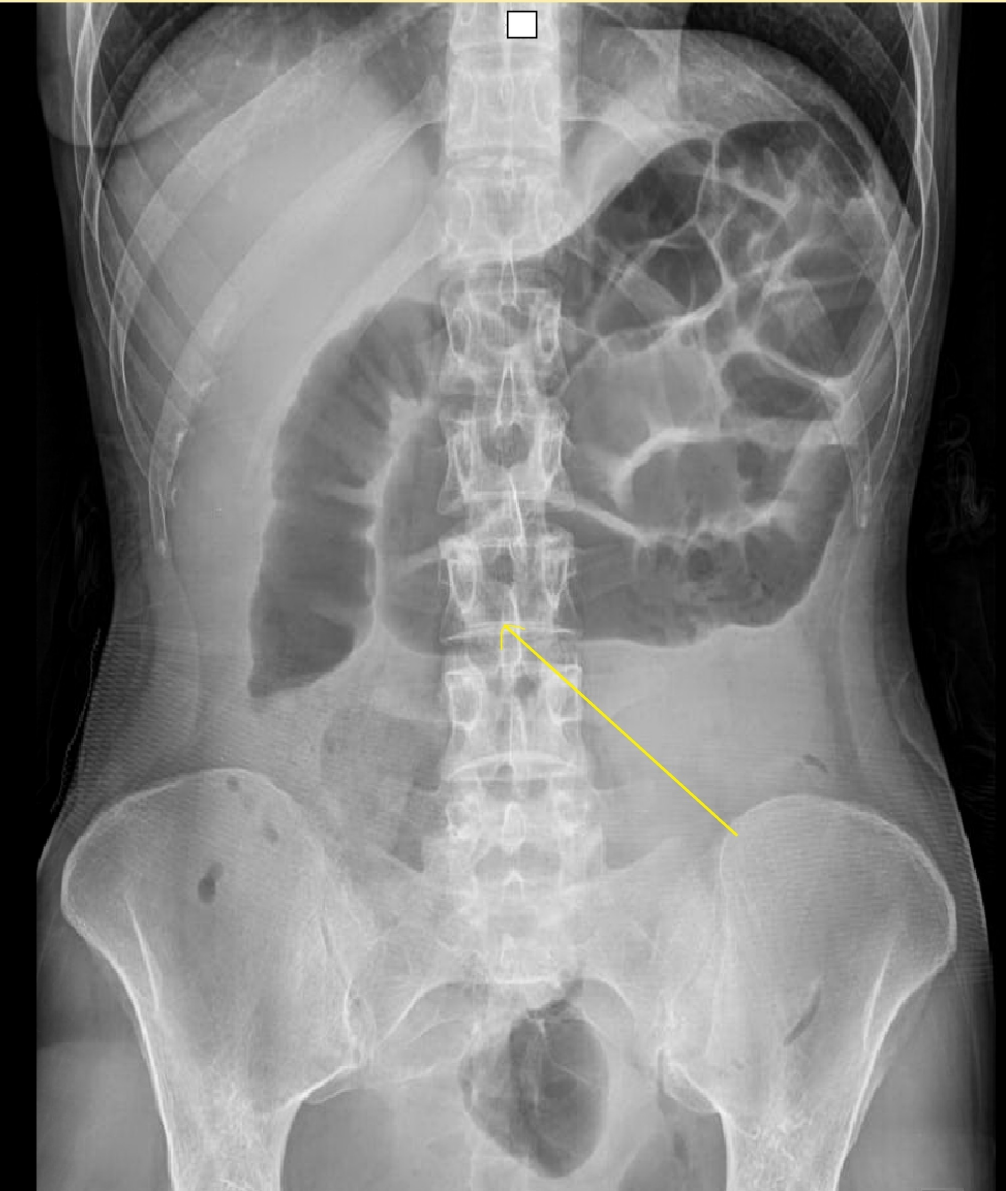
Supine abdominal X-ray A film was taken on admission for complaints of periumbilical pain and diarrhea. This was less than 18 hours before further radiological imaging. The yellow arrow shows the possible junction of the pathological couplet.

A 27-year-old, 43-kg British woman presented to the emergency department with a four-day history of yellow-colored diarrhea followed by vomiting and periumbilical cramping abdominal pain. Eight years prior, the patient had been investigated by gastroscopy only for iron deficiency anemia, but no other investigations were done. Initial investigations showed no signs of sepsis, yet a plain abdominal X-ray showed small bowel obstruction in a surgically virgin abdomen in Figure 1.

**Figure 2:**
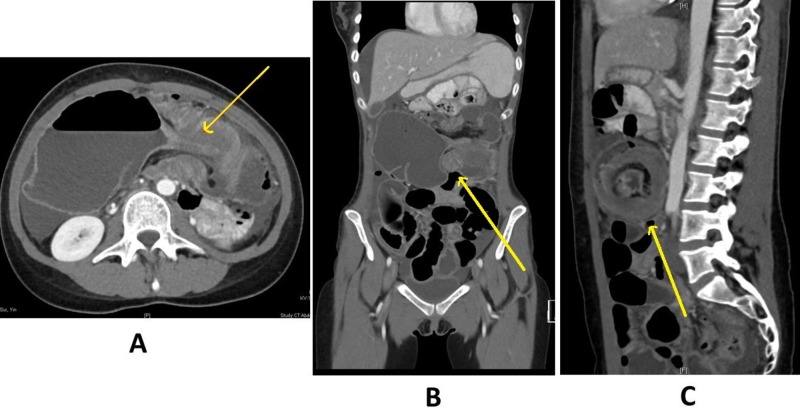
CT scan of pathological couplet Contrast-enhanced abdominal computed tomography (CT) scan showing the combined colocolic intussusception and cecal bascule in the (A) axial, (B) coronal, and (C) sagittal planes. The yellow arrow shows the lead point in the different planes.

As the patient had mild symptoms and was very reluctant for operative intervention, a period of non-operative management was commenced, involving observation overnight, and had a contrast-enhanced abdominal computed tomography (CT) scan the next morning. The formal reporting of the next-day CT scan stated that the patient had a 9 cm cecum “representing either a volvulus or congenital malrotation pulled to the right upper abdomen...in keeping with enteritis or inflammatory bowel disease” in Figure 2.

**Figure 3:**
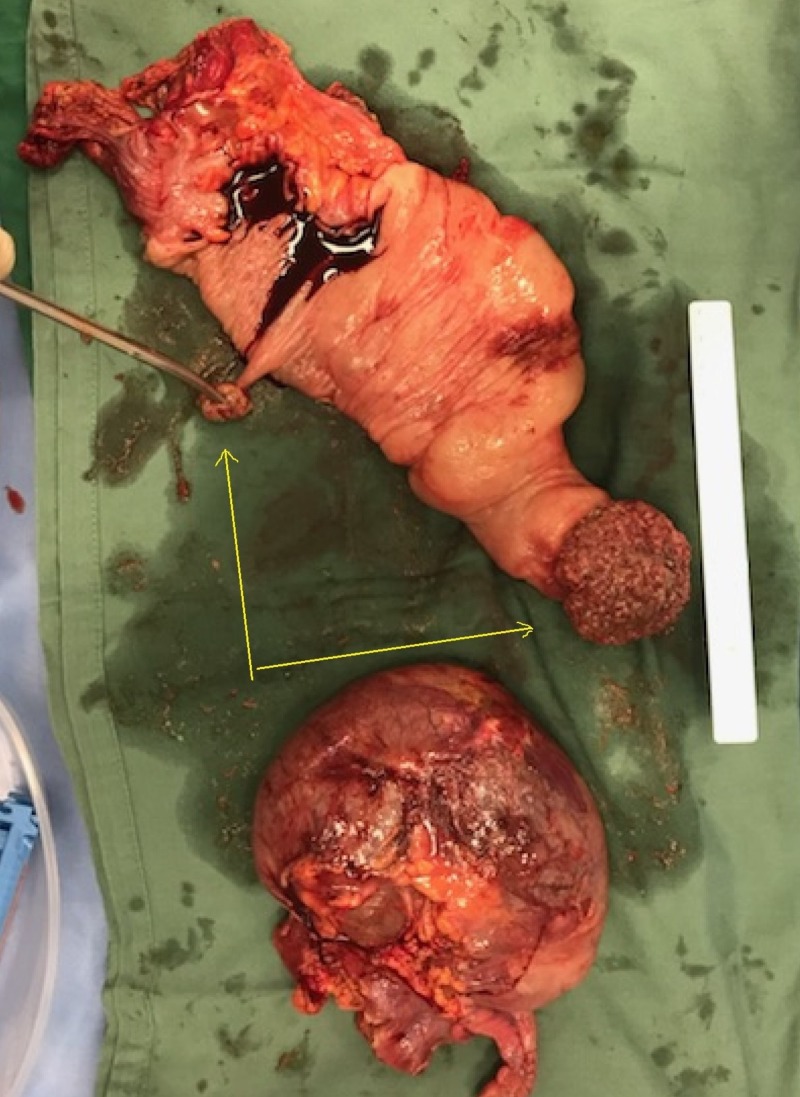
Operative specimen The opened specimen positioned next to a 15 cm ruler. The yellow arrows point to a large sessile polyp as the lead point and to a smaller pedunculated polyp. The bottom specimen is the necrotic cecal bascule.

**Figure 4:**
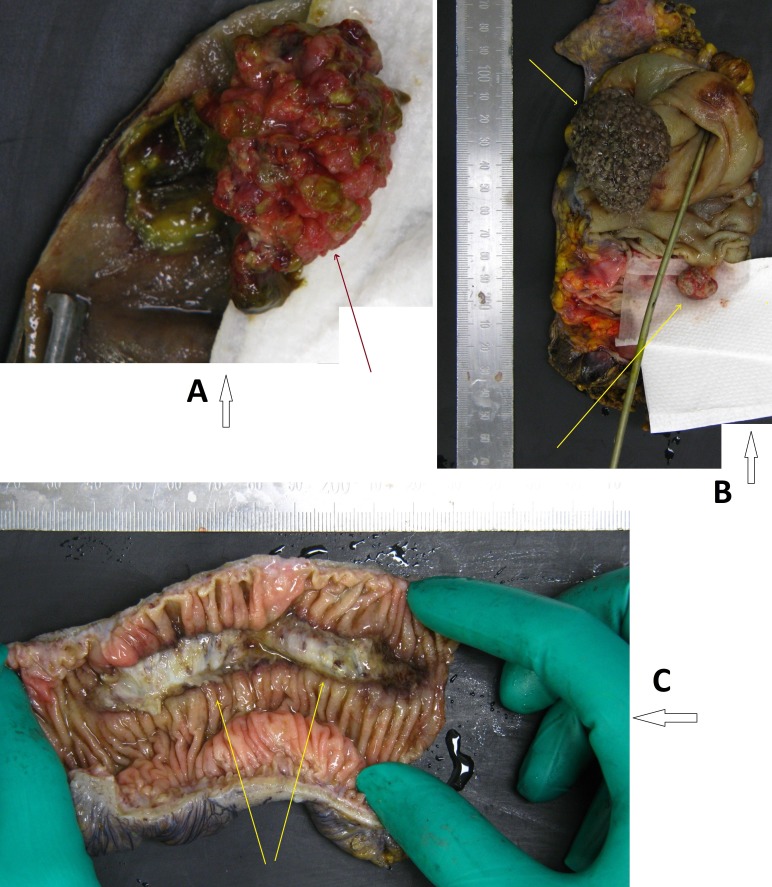
Anatomical pathology specimens The arrows show the specimens and additional findings as examined by the pathologists: (A) tubulovillous adenoma in the transverse colon; (B) lead-point tubular adenoma causing the intussusception and a synchronous tubulovillous adenoma in the ascending colon; and (C) two ischaemic areas in the lateral wall of the descending colon.

The patient underwent an emergent diagnostic laparoscopy with plans for a right hemicolectomy, which was quickly converted to a midline laparotomy incision due to poor intraperitoneal vision from a markedly distended cecum. A necrotic distended cecal bascule with a sealed subhepatic perforation was found, thus a stapled cecectomy across the twist was done to prevent the intravascular release of toxic metabolites and to allow uncrowded access into the abdominal cavity. Other findings were an inflamed mid-ascending colocolic intussusception and two unexplained areas of ischaemic ulceration on the lateral walls of the descending colon. An initial abbreviated laparotomy was done to excise these pathological areas of the colon. The intussusceptum's lead-point is seen in Figure 3. At 48 hours, the patient underwent a relook laparotomy for subtotal colectomy with a diverting loop ileostomy and was eventually was discharged from hospital on Day 20. A formal referral was organized to the geneticist to determine the possibility of a predisposing polyposis syndrome in this young, adult female patient. This is the first reported case to describe this highly rare "pathological couplet." Investigations for possible prothrombotic states returned as negative and were thus unhelpful in explaining the cause of the ischaemic ulceration in the lateral aspect of the descending colon. Figure 4 shows the gross anatomical specimens described by the pathologists.

## Discussion

There are several lessons to be learned from this case such that a different approach could be considered if a similar case were to present at the hospital in the future. These key points are making the diagnosis of either condition individually or in tandem; deciding on conservative versus operative management options; the timing and choosing of investigations and operative interventions; understanding the cause of the pathological couplet in this patient; explaining the cause of concomitant ischaemic ulcerations in the descending colon; and deciding the extent of colonic resection during the initial operation.

Diagnosing a large bowel obstruction in a young adult is very straightforward but concluding from the historical and clinical examination that it is due to either an intussusception or a cecal bascule is tough. Diagnosing this pathological couplet in the same way would be doubly challenging. The unusual clinical presentation of this case would be abnormal in any patient age group and especially unexpected in a young adult with minimal specific clinical signs. In this case, the clinical features of currant jelly stools or a palpable mass were absent. If a similar patient is clinically stable, graduated radiological imaging with an abdominal ultrasound scan, abdominal X-ray, or contrast-enhanced CT scanning would be the mainstay of determining the diagnosis of either an intussusception or a cecal bascule. In this patient with a virgin abdomen, an argument could be made for diagnostic laparoscopy as the sole diagnostic tool.

Timing and choosing the right intervention are challenging in unusual cases, thus an argument for a more initial aggressive approach could be made. In retrospect, an earlier operative intervention would have been better for this patient, as it may have prevented the cecal perforation. The factors of the patient’s condition, patient’s choice, and presumed enteritis delayed this choice. Numerous textbooks describe less invasive hydrostatic or pneumatic techniques to reduce either an intussusception or a cecal bascule mainly for use in pediatric patient populations. The clinical course in this reported case excluded any consideration of non-operative management, which followed the findings of Khan and colleagues who estimated that 70%-90% of adults with intussusception undergo a surgical resection [9]. An earlier laparoscopic approach could have been feasible, however, the worsening cecal distension that occurred during the short 24-hour operative delay forced an open approach. Choosing an early operative intervention quickly after a confirmed or suspected diagnosis has been associated with no mortalities in numerous case reports of adult cecal bascule or colocolic intussusception.

Trying to determine the cause of this rare couplet was a daunting task and, in some instances, the diagnoses are made only at the time of operative intervention. Shah et al. recently published a case of splenic colocolic intussusception in a young man due to hamartomas of Peutz-Jeghers polyposis [10]. When an intussusception does occur in a younger adult, it appears to be more related to a polyp disease than to a malignant tumor in older patients. Regarding the pathological couplet in the case report here, intussusception is suspected to have been the sentinel event that led to a cecal closed-loop obstruction with a competent ileocecal valve that oddly progressed to a volvulus due to a mild element of congenital cecal malposition. Further speculation is proposed regarding the occurrence of two areas of ischaemic ulceration in the lateral wall of the descending colon. The first hypothesis was that it was due to a prothrombotic state, which was not confirmed despite investigations done during this admission. The second hypothesis is that these ischaemic ulcerations resulted from thromboembolizations originating at the left colic artery and/or from the outflow obstruction of the superior mesenteric vein. These concomitant events may have occurred from vascular kinking during stretching, partial twisting, and the resultant pulling up of the mesocolon of the cecum and ascending colon.

Lastly, deciding on the extent of colonic resection is contentious. In this case, it was decided for a subtotal colectomy with a future ileorectal anastomosis to minimize the future risk of colorectal cancer for this yet-to-be-identified juvenile, polyp-forming condition. A brief consideration for preserving the externally normal-appearing, well-vascularised transverse colon for improved fecal continence. Fortuitously, this was realized as being unwise after a sizeable polyp was found post-excision. This dilemma could be assisted in the future with an intraoperative endoscopic evaluation of any segment of bowel considered for preservation.

## Conclusions

This is a very rare case involving a patient where every aspect was uncommon: young, female, a couplet of intussusception and cecal bascule, colocolic type, and a benign pathology as the lead-point. In possible, similar future cases, we would be inclined and hence endorse an early operative approach, possibly avoiding any radiological investigation, thus proceeding to diagnostic laparoscopy involving the resection of all obvious diseased colonic segments. Having such an abnormal pathological couplet often widens the extent of the surgical resection that is performed, but this invariably negates all consideration of simpler methods, such as the pneumatic or manual reduction of an intussusception or the pexying of a cecal bascule or volvulus.

## References

[1] Wilson A, Elias G, Dupiton R (2013). Adult colocolic intussusception and literature review. Case Rep Gastroenterol.

[2] Barussaud M, Regenet N, Briennon X (2006). Clinical spectrum and surgical approach of adult intussusceptions: a multicentric study. Int J Colorectal Dis.

[3] Gayer G, Apter S, Hofmann C, Nass S, Amitai M, Zissin R, Hertz M (1998). Intussusception in adults: CT diagnosis. Clin Radiol.

[4] Hasbahceci M, Basak F, Alimoglu O (2012). Cecal volvulus. Indian J Surg.

[5] Lung BE, Yelika SB, Murthy AS, Gachabayov M, Denoya P (2018). Cecal bascule: a systematic review of the literature. Tech Coloproctol.

[6] Bobroff LM, Messinger NH, Subbarao K, Beneventano TC (1972). The cecal bascule. Am J Roentgenol Radium Ther Nucl Med.

[7] Ruiz de la Hermosa A, Ortega-Domene P, Fuenmayor-Valera ML, Perez-Morera A, Seoane-Gonzalez JB (2016). Caecal bascule, an unusual cause of intestinal obstruction [Article in Spanish, English]. Cir Cir.

[8] Delabrousse E, Sarlieve P, Sailley N, Aubry S, Kastler BA (2007). Cecal volvulus: CT findings and correlation with pathophysiology. Emerg Radiol.

[9] Khan Z, Darr U, Renno A, Alkully T, Rafiq E, Sodeman T (2017). Transient descending colocolonic intussusception due to a large fecaloma in an adult. ACG Case Rep J.

[10] Shah J, Sunkara T, Xiao P, Gaduputi V, Reddy M, Razia S (2018). Peutz-Jeghers syndrome presenting as colonic intussusception: a rare entity. Gastroenterology Res.

